# An error analysis for image-based multi-modal neural machine translation

**DOI:** 10.1007/s10590-019-09226-9

**Published:** 2019-04-08

**Authors:** Iacer Calixto, Qun Liu

**Affiliations:** 1 University of Amsterdam, ILLC, Science Park, Amsterdam, Netherlands; 2 Huawei Noah’s Ark Lab, Hong Kong, Hong Kong

**Keywords:** Multi-modal machine translation, Machine translation, Error analysis, Neural machine translation, Multi-modal neural machine translation

## Abstract

In this article, we conduct an extensive quantitative error analysis of different multi-modal neural machine translation (MNMT) models which integrate visual features into different parts of both the encoder and the decoder. We investigate the scenario where models are trained on an in-domain training data set of parallel sentence pairs with images. We analyse two different types of MNMT models, that use *global* and *local* image features: the latter encode an image globally, i.e. there is one feature vector representing an entire image, whereas the former encode spatial information, i.e. there are multiple feature vectors, each encoding different portions of the image. We conduct an error analysis of translations generated by different MNMT models as well as text-only baselines, where we study how multi-modal models compare when translating both *visual and non-visual terms*. In general, we find that the additional multi-modal signals consistently improve translations, even more so when using simpler MNMT models that use global visual features. We also find that not only translations of terms with a strong visual connotation are improved, but almost all kinds of errors decreased when using multi-modal models.

## Introduction

Neural machine translation (NMT) has recently been successfully tackled as a sequence to sequence (*seq2seq*) learning problem (Kalchbrenner and Blunsom 2013; Cho et al. 2014; Sutskever et al. 2014). In this problem, each training example consists of one source and one target variable-length sequence, and there is no prior information regarding the alignments between the two. A model is trained to *translate* sequences *X* in the source language into their corresponding translations *Y* in the target. This framework has been successfully used in many different tasks related to natural language processing, such as handwritten text generation (Graves 2013), image description generation (Hodosh et al. 2013; Kiros et al. 2014; Mao et al. 2014; Vinyals et al. 2015; Elliott et al. 2015; Karpathy and Fei-Fei 2015), MT (Cho et al. 2014; Sutskever et al. 2014), and video description generation (Donahue et al. 2015; Venugopalan et al. 2015).

Multi-modal MT (MMT) is an exciting novel take on MT where we are interested in learning to *translate sentences in the context of images*. The main goal in MMT is to learn models that can exploit visual information, in other words have *visually-grounded* (Harnad 1990; Glenberg and Robertson 2000) MT models trained on sentences and images. MT models grounded on visual inputs are expected to learn to better handle certain types of ambiguous sentences by exploiting *visual context* (Calixto et al. 2012). To mention two rather trivial examples of ambiguity: “The beautiful jaguar is really fast” has an ambiguous noun phrase, and the textual context (“is really fast”) cannot really help disambiguate it; or the classical “The man on the hill saw the boy with a telescope”, which can knowingly have many different interpretations (Church and Patil 1982). In both examples, having an image illustrative of the sentence could be the additional signal that enables the model to arrive at the correct sentence interpretation and ultimately translation.

In the last 2 years there have been two shared tasks (Specia et al. 2016; Elliott et al. 2017) where many research groups proposed different techniques to integrate visual information into MT, e.g. Caglayan et al. (2016) and Libovický and Helcl (2017).

This work aims to provide a comprehensive quantitative error analysis of translations generated with different variants of multi-modal NMT (MNMT) models, more specifically the MNMT models introduced in Calixto et al. (2017) and Calixto and Liu (2017).^1^ The main contributions of our work are:

- We conduct a comprehensive error analysis on the translations of image descriptions generated by different MNMT models, comparing these models to other MNMT models as well as to two strong text-only baselines: a phrase-based statistical MT model (PBSMT; Koehn et al. 2003), and a text-only attention-based NMT model (Bahdanau et al. 2015; Luong et al. 2015).
- We establish that MNMT models are better at translating sentences in the presence of an image when these sentences are image descriptions.
- We show that MNMT models consistently improve translations of not only terms with a clear visual interpretation, but also of other more general error types without a direct visual connotation.

The remainder of this article is structured as follows. In Sect. 2 we discuss relevant previous related work. We then revise the attention-based NMT framework (Sect. 3) and expand it to briefly introduce the different MNMT models proposed in Calixto et al. (2017) and Calixto and Liu (2017) (Sect. 3.2). In Sect. 4 we introduce the data sets we used in our evaluation. In Sect. 5 we report on a quantitative error analysis of the different models discussed in this work, as well as discuss and interpret our main findings. Finally, in Sect. 6 we draw conclusions and provide avenues for future work.

## Related work

MMT has only recently been addressed by the MT community in the form of a shared task (Specia et al. 2016; Elliott et al. 2017). However, there is a vast amount of previous work where researchers tried to incorporate non-textual signals to train multi-modal models of language (Farhadi et al. 2010; Silberer and Lapata 2012; Kiros et al. 2014; Mao et al. 2014; Chen et al. 2017; Faghri et al. 2017). We also highlight that the use of images has been widely studied in the context of training multi-modal word representations (Bruni et al. 2014; Lazaridou et al. 2015; Mao et al. 2016).

More similarly to our task, there has been considerable work on using images in tasks involving multi-modal (and sometimes multilingual) *natural language generation*, such as in image captioning (Vinyals et al. 2015; Xu et al. 2015) and visual question answering (Gao et al. 2015; Wu et al. 2017). We now discuss how different researchers proposed to incorporate visual information specifically into MT, and categorise different research efforts in whether authors utilise global or spatial visual features in multi-modal translation models, and whether they use a multi-task learning approach.

### MMT models using global visual features

Calixto et al. (2012) first studied how the visual context of a textual description can be helpful in the disambiguation of SMT systems. Elliott et al. (2015) generated multilingual descriptions of images by learning and transferring features between two independent neural image description models. Although not an NMT model, Hitschler et al. (2016) used image features to re-rank translations of image descriptions generated by an SMT model and reported significant improvements. To the best of our knowledge, Hitschler et al. (2016) and Luong et al. (2016) were the first to utilise image features to somehow improve MT.

More recently, different research groups have proposed to include visual features directly into NMT models with some success (Caglayan et al. 2017; Elliott and Kádár 2017; Madhyastha et al. 2017). We note that in the official results of the first MMT shared task (Specia et al. 2016) no submissions based on a purely neural architecture improved on the PBSMT baseline. Nevertheless, researchers have proposed to include global visual features in re-ranking *n*-best lists generated by a PBSMT system or directly in a purely NMT framework with varied degrees of success (Caglayan et al. 2016; Calixto et al. 2016; Libovický et al. 2016; Shah et al. 2016). The best results achieved by a purely NMT model at that stage were those of Huang et al. (2016), who proposed to use global and regional image features extracted with the VGG19 (Simonyan and Zisserman 2014) and the RCNN (Girshick et al. 2014) convolutional neural networks (CNNs), respectively. Huang et al. (2016) extract global features for an image, project these features into the vector space of the source words and then add them as a word in the input sequence. Their best model improves over a strong NMT baseline, but is not significantly better than a PBSMT baseline trained on the same data.

Their model is similar to that of Calixto and Liu (2017), the main differences being that in Calixto and Liu (2017) image features are included separately either as a word in the source sentence (Sect. 3.2.1) or *directly* for encoder (Sect. 3.2.2) or decoder initialisation (Sect. 3.2.3), whereas Huang et al. (2016) only use it as a word. Calixto and Liu (2017) also show that it is better to include an image exclusively for the encoder *or* the decoder initialisation, but not both.

Finally, Caglayan et al. (2017) proposed to interact image features with target word embeddings, more specifically to perform an element-wise multiplication of the (projected) global image features and the target word embeddings before feeding the target word embeddings into their decoder recurrent neural network. They reported significant improvements by using image features to gate target word embeddings and won the 2017 MMT shared task (Elliott et al. 2017).

### MMT models using local visual features

Recently, different research groups have used *local* or *spatial visual features* in an encoder–decoder NMT framework to incorporate images into their model (Calixto et al. 2017; Libovický and Helcl 2017; Caglayan et al. 2017). Local visual features encode different areas of an image separately in different feature vectors by using the activations of different layers of a CNN. This can be interesting since these features can be effectively incorporated via an attention mechanism over the image representations.

However, using spatial visual features have some drawbacks one needs to account for. Specifically, local features are considerably larger than their global counterparts. For instance, if we use the VGG19 network (Simonyan and Zisserman 2014) to extract local and global features for one same image, local features (i.e., layer CONV5, 4) consist of a $196 \times 1024$D matrix, whereas global features (i.e., layer FC7) consist of a 4096D vector. That is an increase of $49\times$ the amount of memory used to store image features, which will have an impact on the training time as well as on the memory footprint of a MNMT model.^2^ Moreover, to use an additional attention mechanism, as proposed by Calixto et al. (2017), means to add more parameters to the NMT model itself, i.e. the authors report an increase in $6.6\%$ in model size. This can become problematic specially if the amount of multi-modal training examples is small.

### Multi-task learning MMT

Finally, another successful approach to MMT involves multi-task learning. Luong et al. (2016) proposed a multi-task approach where a model is trained using two tasks and a shared decoder: the main task is to translate from German into English and the secondary task is to generate English descriptions given an image. They show improvements in the main translation task when also training for the secondary image description task. More recently, Elliott and Kádár (2017) propose a multi-task learning model trained to do translation (English $\rightarrow$ German) and sentence–image ranking (English $\leftrightarrow$ image), using a standard word cross-entropy and margin-based losses as its task objectives, respectively. Their model uses the pre-trained GoogleNet v3 CNN (Szegedy et al. 2016) and pool5 features.

We now move on to introduce the baseline NMT and the MNMT models used in this work.

## Attention-based text-only and multi-modal NMT

We first briefly introduce our attention-based NMT baseline, and move on to describe the MNMT models of Calixto and Liu (2017) (Sects. 3.2.1, 3.2.2, 3.2.3) and Calixto et al. (2017) (Sect. 3.2.4).

### Neural machine translation

Given a source sequence ${X = (x_1, \ldots , x_N)}$ and its translation ${Y = (y_1, \ldots , y_M)},$ a standard NMT model is a single neural network that translates *X* into *Y* by directly learning to compute $p(Y \,\vert \,X).$ Each $x_i$ is a row index in a source lookup matrix ${\varvec{W}}_x \in \mathbb {R}^{|V_x| \times d_x}$ (the *source word embeddings matrix*) and each $y_j$ is an index in a target lookup matrix ${\varvec{W}}_y \in \mathbb {R}^{|V_y| \times d_y}$ (the *target word embeddings matrix*). $V_x$ and $V_y$ are source and target vocabularies and $d_x$ and $d_y$ are source and target word embeddings dimensionalities, respectively.

The encoder is a bidirectional RNN (Schuster and Paliwal 1997) with GRU units (Cho et al. 2014), where a forward RNN $\overrightarrow{\varPhi }_{\text {enc}}$ reads *X* from left to right and computes *forward annotation vectors*${(\overrightarrow{{\varvec{h}}}_1, \ldots , \overrightarrow{{\varvec{h}}}_N)},$ and a backward RNN $\overleftarrow{\varPhi }_{\text {enc}}$ reads *X* from right to left computing *backward annotation vectors*${(\overleftarrow{{\varvec{h}}}_1, \ldots , \overleftarrow{{\varvec{h}}}_N)},$ as in (1):

1 $$\begin{aligned} \overrightarrow{{\varvec{h}}_i}= & {} \overrightarrow{\varPhi }_{\text {enc}} \big ( {\varvec{W}}_x[x_i], \overrightarrow{{\varvec{h}}}_{i-1} \big ), \nonumber \\ \overleftarrow{{\varvec{h}}_i}= & {} \overleftarrow{\varPhi }_{\text {enc}} \big ( {\varvec{W}}_x[x_i], \overleftarrow{{\varvec{h}}}_{i+1} \big ), \nonumber \\ {\varvec{h}}_i= & {} \big [ \overrightarrow{{\varvec{h}}_i}; \overleftarrow{{\varvec{h}}_i} \big ], \end{aligned}$$

where ${\varvec{h}}_i$ is the final annotation vector corresponding to word $x_i.$ The decoder is an RNN with GRU conditioned on the previously emitted words and the source sentence annotation vectors via a Bahdanau-style (i.e. a one-layer MLP) attention mechanism (Bahdanau et al. 2015). We denote decoder hidden states by ${\varvec{s}},$ and by ${\varvec{s}}_t$ when referring to a specific decoder time-step.

In the attention mechanism first a single-layer MLP is used to compute an *expected alignment*$e^\text {src}_{t,i}$ between each source annotation ${\varvec{h}}_i$ and the target word ${\hat{y}}_t$ to be emitted at the current time step *t*. A time-dependent attention vector ${\varvec{c}}_t$ is computed as a weighted sum over the source annotation vectors as shown in (2)–(4):

2 $$\begin{aligned} e^\text {src}_{t,i}&= ({\varvec{v}}^\text {src}_a)^T \tanh ( {\varvec{U}}^\text {src}_a {\varvec{s}}'_{t} + {\varvec{W}}^\text {src}_a {\varvec{h}}_i),\end{aligned}$$

3 $$\begin{aligned} \alpha ^\text {src}_{t,i}&= \frac{\exp {(e^\text {src}_{t,i})}}{ \sum _{j=1}^{N}{\exp {(e^\text {src}_{t,j})}} }, \end{aligned}$$

4 $$\begin{aligned} {\varvec{c}}_t&= {\sum _{i=1}^{N}{ \alpha ^\text {src}_{t,i} {\varvec{h}}_i }}, \end{aligned}$$

where $$\alpha ^\text {src}_{t,i}$$ is the normalised alignment matrix between each source annotation vector $${\varvec{h}}_i$$ and the word $${\hat{y}}_t$$ to be emitted at time step *t*,  and $${\varvec{v}}^\text {src}_a,$$$${\varvec{U}}^\text {src}_a$$ and $${\varvec{W}}^\text {src}_a$$ are model parameters. $${\varvec{s}}'_t$$ is a *candidate hidden state* computed based on the previous decoder hidden state $${\varvec{s}}_{t-1}$$ and the previously emitted word $${\hat{y}}_{t-1}.$$^3^

Finally, the initial decoder hidden state ${\varvec{s}}_0$ is computed using a single-layer feed-forward neural network. It uses the concatenation of the last hidden states of the encoder forward and backward RNNs, as in (5):

5 $$\begin{aligned} {\varvec{s}}_0 = \tanh \big ( {\varvec{W}}_{di} [\overleftarrow{{\varvec{h}}}_1; \overrightarrow{{\varvec{h}}_N}] + {\varvec{b}}_{di} \big ), \end{aligned}$$

where ${\varvec{W}}_{di}$ and ${\varvec{b}}_{di}$ are model parameters.

### Multi-modal NMT (MNMT)

The models discussed in this section can be seen as extensions of the standard attention-based NMT framework with the addition of a *visual component* to incorporate image features. These models use pre-trained CNNs to extract visual features for all images, where these features can be *global* features that describe the entire image, or *spatial* features that describe different portions of the image separately.

Global visual features ${\varvec{q}}$ are obtained from the 19-layer VGG19 network (Simonyan and Zisserman 2014), and are the 4096D activations of the penultimate fully-connected layer FC7. Spatial visual features ${\varvec{v}}_{\text {S}}$ are obtained from the 50-layer ResNet-50 (He et al. 2015), and are the activations of the $\texttt {res4f}$ layer. They can be seen as encoding an image in a $14\times 14$ grid, where each of the entries in the grid is represented by a 1024*D* feature vector that only encodes information about that specific region of the image. The final features consist of the $14\times 14$ grid linearised into a $196 \times 1024$ matrix ${\varvec{v}}_{\text {S}} = ({\varvec{a}}_1, {\varvec{a}}_2, \ldots , {\varvec{a}}_L), {\varvec{a}}_l \in \mathbb {R}^{1024}$ where each of the $L = 196$ rows consists of a 1024D feature vector and each column, i.e. feature vector, represents one grid in the image. Both CNNs, VGG19 and ResNet-50, are publicly available and were pre-trained for classifying images into one out of the 1000 classes in ImageNet (Russakovsky et al. 2015).

We now briefly refer to the three MNMT models introduced in Calixto and Liu (2017) (Sects. 3.2.1, 3.2.2, 3.2.3), and to the MNMT model proposed in Calixto et al. (2017) (Sect. 3.2.4). For detailed information about these models, we refer the reader to the original publications.

#### Images as source words: $\hbox {IMG}_{\text {2W}}$

In model $\hbox {IMG}_{\text {2W}},$ image features are simply projected into the space of the source word embeddings and then incorporated as the first and last “words” of the source sentence. The encoder RNN reads this source sequence with the additional “image-words”, and the attention mechanism learns when to attend to the image representation similarly as when to attend to other words.

**Fig. 1:**
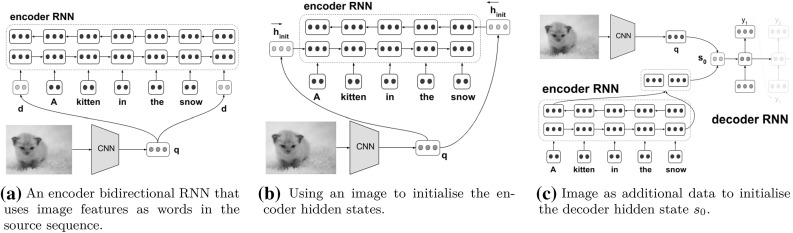
Multi-modal neural machine translation models $$\hbox {IMG}_{\text {W}},$$$$\hbox {IMG}_{\text {E}},$$ and $$\hbox {IMG}_{\text {D}}$$ (Calixto and Liu 2017)

Given a global image feature vector ${\varvec{q}} \in \mathbb {R}^{4096}$:

6 $$\begin{aligned} {\varvec{d}} = {\varvec{W}}_I^2 \cdot ( {\varvec{W}}_I^1 \cdot {\varvec{q}} + {\varvec{b}}_I^1 ) + {\varvec{b}}_I^2 , \end{aligned}$$

where ${\varvec{W}}_I^1 \in \mathbb {R}^{4096 \times 4096}$ and ${\varvec{W}}_I^2 \in \mathbb {R}^{4096 \times d_x}$ are image transformation matrices, ${\varvec{b}}_I^1 \in \mathbb {R}^{4096}$ and ${\varvec{b}}_I^2 \in \mathbb {R}^{d_x}$ are bias vectors, and $d_x$ is the source words vector space dimensionality, all trained with the model. ${\varvec{d}}$ is directly used as a word in the source words vector space as the first and last words of the source sentence. An illustration of this idea is given in Fig. 1a, where a source sentence that originally contained *N* tokens, after including the image as source words will contain $N+2$ tokens.

#### Images for encoder initialisation: $\hbox {IMG}_{\text {E}}$

Two single-layer feed-forward neural networks are used to compute the initial states of the forward and backward RNNs $\overrightarrow{\varPhi }_{\text {enc}}$ and $\overleftarrow{\varPhi }_{\text {enc}},$ respectively, as illustrated in Fig. 1b. Similarly to 3.2.1, given a global image feature vector ${\varvec{q}} \in \mathbb {R}^{4096},$ we compute a vector ${\varvec{d}}$ using Eq. (6), only this time the parameters ${\varvec{W}}_I^2$ and ${\varvec{b}}_I^2$ project the image features into the same dimensionality as the encoder RNN hidden states [as in (1)].

The feed-forward networks used to initialise the encoder hidden state are computed as in (7):

7 $$\begin{aligned} \overleftarrow{{\varvec{h}}}_{\text {init}}&= \tanh \big ( {\varvec{W}}_{f}{\varvec{d}} + {\varvec{b}}_{f} \big ),\nonumber \\ \overrightarrow{{\varvec{h}}}_{\text {init}}&= \tanh \big ( {\varvec{W}}_{b}{\varvec{d}} + {\varvec{b}}_{b} \big ), \end{aligned}$$

where ${\varvec{W}}_{f}$ and ${\varvec{W}}_{b}$ are multi-modal projection matrices that project the image features ${\varvec{d}}$ into the encoder forward and backward hidden states dimensionality, respectively, and ${\varvec{b}}_{f}$ and ${\varvec{b}}_{b}$ are bias vectors.

#### Images for decoder initialisation: $\hbox {IMG}_{\text {D}}$

In model $\hbox {IMG}_{\text {D}},$ illustrated in Fig. 1c, image features are incorporated as additional input to initialise the decoder hidden state at time step $t=0,$ as in (8):

8 $$\begin{aligned} {\varvec{s}}_0 = \tanh \big ( {\varvec{W}}_{di} [\overleftarrow{{\varvec{h}}}_1; \overrightarrow{{\varvec{h}}_N}] + {\varvec{W}}_{m}{\varvec{d}} + {\varvec{b}}_{di} \big ), \end{aligned}$$

where ${\varvec{W}}_{m}$ is a multi-modal projection matrix that maps the image features ${\varvec{d}}$ onto the decoder hidden state dimensionality and ${\varvec{W}}_{di}$ and ${\varvec{b}}_{di}$ are model parameters. Once again, ${\varvec{d}}$ is computed as in Eq. (6), only this time the parameters ${\varvec{W}}_I^2$ and ${\varvec{b}}_I^2$ project the image features into the same dimensionality as the decoder hidden states.

#### Visual attention mechanism: $\hbox {NMT}_{\text {SRC+IMG}}$

**Fig. 2:**
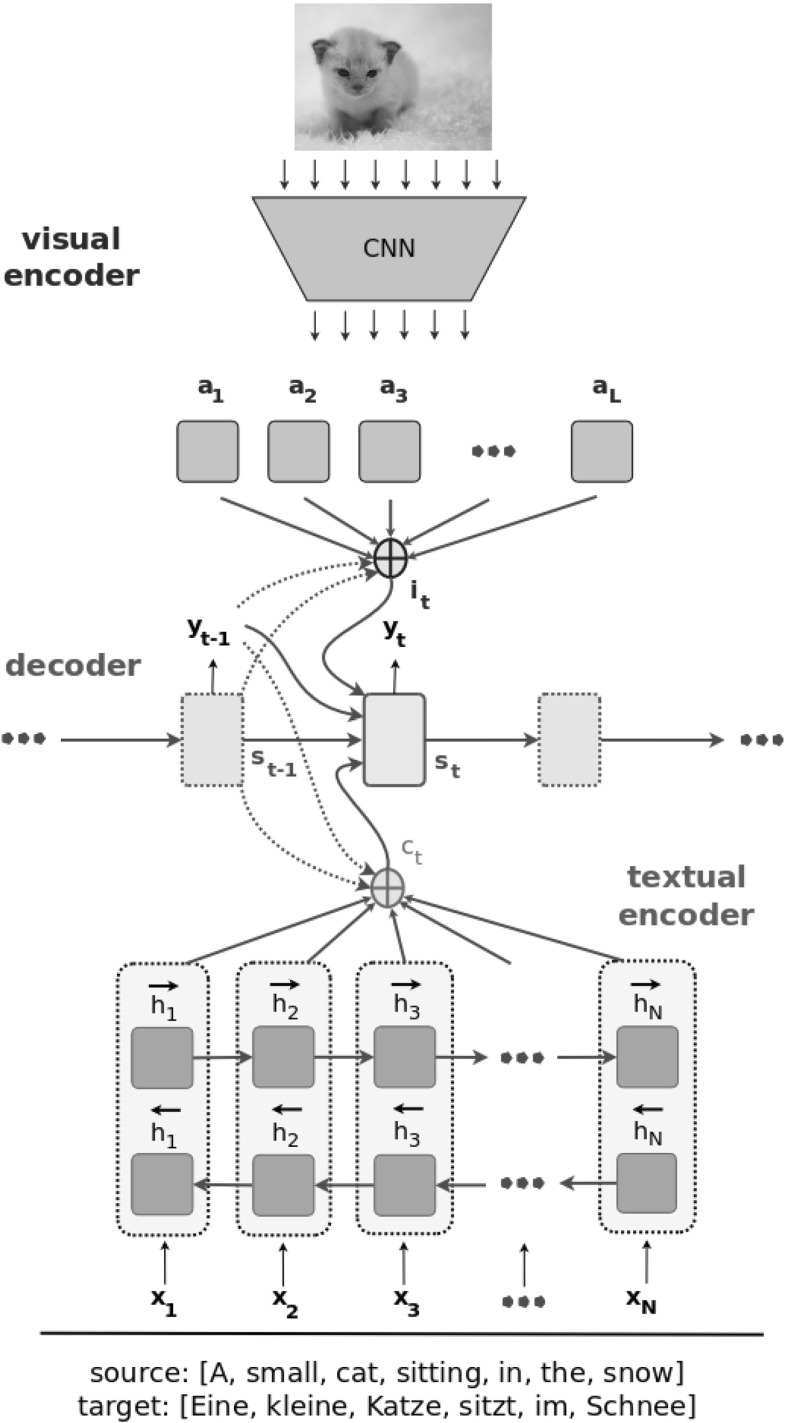
A doubly-attentive decoder learns to attend to image patches and source-language words independently when generating translations (Calixto et al. 2017)

The last MNMT model analysed in this work is the doubly-attentive MNMT model introduced in Calixto et al. (2017) and illustrated in Fig. 2. It incorporates spatial visual features by means of an independent visual attention mechanism, which is implemented similarly to the source-language attention mechanism (the MLP attention of Bahdanau et al. 2015; Luong et al. 2015), as in (9):

9 $$\begin{aligned} e^\text {img}_{t,l}&= ({\varvec{v}}^\text {img}_a)^T \tanh ( {\varvec{U}}^\text {img}_a {\varvec{s}}'_{t} + {\varvec{W}}^\text {img}_a {\varvec{a}}_l),\nonumber \\ \alpha ^\text {img}_{t,l}&= \frac{\exp {(e^\text {img}_{t,l})}}{ \sum _{j=1}^{L}{\exp {(e^\text {img}_{t,j})}}},\nonumber \\ \beta _t&= \sigma ( {\varvec{W}}_{\beta } {\varvec{s}}_{t-1} + {\varvec{b}}_{\beta }),\nonumber \\ {\varvec{i}}_t&= \beta _t \sum _{l=1}^{L}{ \alpha ^\text {img}_{t,l} {\varvec{a}}_l }, \end{aligned}$$

where $$\alpha ^\text {img}_{t,l}$$ is the normalised alignment matrix between all the image feature vectors $${\varvec{a}}_l$$ and the target word to be emitted at time step *t*,  and $${\varvec{v}}^\text {img}_a,$$$${\varvec{U}}^\text {img}_a,$$$${\varvec{W}}^\text {img}_a,$$$${\varvec{W}}_{\beta },$$ and $${\varvec{b}}_{\beta }$$ are model parameters. $$\beta _t \in [0,1]$$ is a gating scalar used to weight the expected importance of the image context vector in relation to the next target word at time step *t*,  and $${\varvec{i}}_t$$ is the final image context vector for the target word at time step *t*.

Finally, the time-dependent image context vector ${\varvec{i}}_t$ is used as an additional input to obtain the final hidden state ${\varvec{s}}_t,$ as in Eq. (10):

10 $$\begin{aligned} {\varvec{z}}_t&= \sigma ({\varvec{W}}^\text {src}_z {\varvec{c}}_t + {\varvec{W}}^\text {img}_z {\varvec{i}}_t + {\varvec{U}}_z {\varvec{s}}'_t),\nonumber \\ {\varvec{r}}_t&= \sigma ({\varvec{W}}^\text {src}_r {\varvec{c}}_t + {\varvec{W}}^\text {img}_r {\varvec{i}}_t + {\varvec{U}}_r {\varvec{s}}'_t),\nonumber \\ \underline{{\varvec{s}}}_t&= \tanh ( {\varvec{W}}^\text {src} {\varvec{c}}_t + {\varvec{W}}^\text {img} {\varvec{i}}_t + {\varvec{r}}_t \odot ({\varvec{U}}{\varvec{s}}'_t)),\nonumber \\ {\varvec{s}}_t&= (1 - {\varvec{z}}_t)\odot \underline{{\varvec{s}}}_t + {\varvec{z}}_t \odot {\varvec{s}}'_t. \end{aligned}$$

## Data set

MNMT models need bilingual sentences accompanied by one or more images as training data. The original Flickr30k data set contains 30K images and 5 English sentence descriptions for each image (Young et al. 2014). We use the Multi30k dataset (Elliott et al. 2016), henceforth referred to as M30k, which is a multilingual expansion of the original Flickr30k. For each of the 30K images in the Flickr30k, the M30k has one of its English descriptions manually translated into German by a professional translator. Training, validation and test sets contain 29K, 1014 and 1K images, respectively, each accompanied by one sentence pair (the original English sentence and its German translation).

We use the scripts in Moses (Koehn et al. 2007) to normalise, truecase and tokenize English and German descriptions and we also convert space-separated tokens into subwords (Sennrich et al. 2016). All MT and MMT models use the same vocabulary, and if sentences in English or German are longer than 80 tokens, they are discarded.

We use the entire M30k training set for training, its validation set for model selection with BLEU, and translate and manually analyse a subset of 50 sentences in the M30k test set. In total, we analyse translations generated by 2 baselines and 6 MNMT models, which sum up to a total of 400 manually analysed translations.

A complete description of the experimental settings including a thorough automatic evaluation using standard MT metrics (Papineni et al. 2002; Snover et al. 2006; Denkowski and Lavie 2014; Popović 2015) can be found in the original publications (Calixto and Liu 2017; Calixto et al. 2017), and these are complementary to the main findings in this article. We now move on to describe the error analysis of the translations generated by the different models discussed in this article.

## Error analysis

We believe it is both interesting and useful to know what specific types of errors the different models proposed in Calixto and Liu (2017) and Calixto et al. (2017) make. For that reason, we conduct an error analysis of translations obtained with text-only baselines as well as their multi-modal models, briefly introduced in Sects. 3 and 3.2. Our goal is to shed light on the reasons why certain models perform better than others and in which particular scenarios, and also to verify whether there are systematic mistakes certain models make.

For instance, one intuitive assumption we make regarding the quality of translations obtained with multi-modal models is that they are better at translating *visual terms*, which we define as the terms in a sentence that have a *strong visual component to their meaning*, or in other words terms that have *a strong alignment to* (*parts of*) *the image* that illustrate the sentence where they appear. These would typically consist of nouns and/or certain adjectives, and we propose that *a single word or phrase should be considered a visual term if it describes one or more entities clearly illustrated in the image*. Some examples include e.g. the colour of an object, a mention to an object, or mentions to animals and people in the image. Moreover, since the Multi30k data set consists of images and their descriptions, there will likely be many terms that fall under the *visual term* category.

In our investigation, we randomly select 50 sentences from the translated Multi30k test set and analyse the translations generated by different models trained on the M30k training set. We evaluate the models $$\hbox {IMG}_{\text {2W}},$$$$\hbox {IMG}_{\text {E}},$$$$\hbox {IMG}_{\text {D}},$$$$\hbox {IMG}_{\text {2W+D}},$$$$\hbox {IMG}_{\text {E+D}},$$ and $$\hbox {NMT}_{\text {SRC+IMG}}.$$ For comparison, we also analyse translations generated by two baselines: one PBSMT and one text-only NMT. Models $$\hbox {IMG}_{\text {2W+D}}$$ and $$\hbox {IMG}_{\text {E+D}}$$ are simply the straightforward combination of individual models. In $$\hbox {IMG}_{\text {2W+D}},$$ global visual features are incorporated both as words in the source sequence as well as in the decoder initialisation; in $$\hbox {IMG}_{\text {E+D}},$$ image features are used to compute the encoder RNN initial states, as well as to initialise the decoder. For more details, we refer the reader to Calixto and Liu (2017) and Calixto et al. (2017). These are all models trained to translate from German into English, and the reason we perform our error analysis on the translations into English is to make it more useful to a broader audience.

### Error taxonomy

We follow previous work and adopt an error taxonomy that is both simple to understand and addresses our needs. Our error taxonomy is adapted from the one introduced in Vilar et al. (2006), with few differences. These differences are mostly due to the fact that we want to measure how our models translate terms that describe concepts that have a direct correspondence in the image, which we refer to as *visual terms*. Additionally, some of the fine-grained distinctions in the taxonomy proposed in Vilar et al. (2006) are not necessary in our work, in which cases we just kept the high-level error type without differentiating further between specific sub-errors. Finally, the possible categories to select from are:

- *Missing words* there are words missing in the translation. These words can be *content words*, which are central to convey the meaning of the sentence, or *filler words*, which are only necessary to make the sentence grammatical. We do not distinguish between missing content and filler words for simplicity, and only report the aggregated number of missing words.
- *Incorrect words* words were incorrectly translated. We distinguish between the following types of incorrect word error types:
- *Mistranslation* includes cases where there is a wrong disambiguation, lexical choice and/or a spurious translation.
- *Incorrect form*, *extra words or style* includes cases where there are spelling mistakes or mistakes in the inflected word, although the base form is correct; some of the source words are translated more than once, i.e. over-translation; and also includes errors where the translation makes sense, i.e. the main sentence meaning is conveyed, but it does not read fluently. For simplicity, we only report the aggregated number for the three types.
- *Other* aggregates other important errors types, more specifically: *word order*, where translations have wrong word order, *unknown words*, where there are parts of the source sentence that were left untranslated, and *punctuation*, where there are wrong punctuation marks.

In order to measure how well different models translate visual terms, we also mark whenever a model translates a visual term correctly and incorrectly. Additionally, we are also interested in the cases where a model can generate “novel” terms by exploiting visual information, i.e. the textual description generated by the model does not have an obvious corresponding mention in the source sentence to align to, but could have been inferred at least partially from the image. Finally, one last case we investigate is when a model translates visual terms incorrectly, but there is something interesting about the mistake made by the model. Interestingness is clearly a subjective quality, and we typically select examples where the translation is wrong but there is a reasonable *visual* explanation for the mistake, e.g. the model translates “Elephant trunk” as “Elephant hose”.

Thus, we add an additional *visual* category to the categories originally proposed in Vilar et al. (2006), with four subcategories:

- *correct* a visual term is correctly translated,
- *mistranslation* a visual term is incorrectly translated,
- *incorrect but interesting* a visual term is incorrectly translated but there is something interesting about the mistake,
- *novel* a visual term is correctly generated without a corresponding mention in the source sentence, meaning that the visual term *could have* been inferred from the image.

### Discussion

In Table 1, we present an error analysis of translations generated by models trained on the M30k training set for 50 randomly selected sentences from the M30k test set.

**Table 1:**
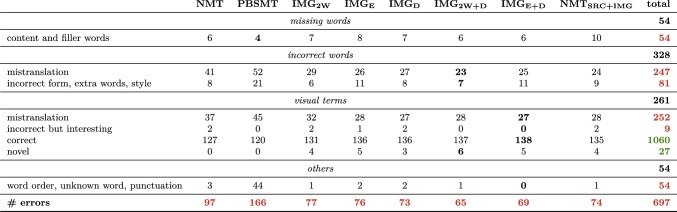
Results of the error analysis of translations obtained for 50 randomly selected sentences from the M30k test set

Models are all trained on the M30k training set. We show the overall quantity of different errors types identified in translations generated by each model. Best results per error type are shown in bold. Subtotals are shown in red when a lower score is better, i.e. the score represents an error, and in green when higher is better, i.e. the score represents a measure of correct predictions

#### Missing words

We start by analysing the missing words category. The behaviour of model $$\hbox {NMT}_{\text {SRC+IMG}}$$ is rather unexpected, since it generates translations with the highest number of missing content and filler words, even more than the baselines. Models $$\hbox {IMG}_{\text {E}},$$$$\hbox {IMG}_{\text {2W}},$$ and $$\hbox {IMG}_{\text {D}}$$ follow next, also presenting more content words missing than the baselines. This indicates that these models suffer the most from the *under-translation* problem, discussed by Tu et al. (2016). The main reason could be that there are not enough training examples to bootstrap attention weights in model $$\hbox {NMT}_{\text {SRC+IMG}},$$ or a good mapping from visual features to word embeddings/encoder/decoder hidden states in the other models. This hypothesis is corroborated by results of models $$\hbox {IMG}_{\text {E+D}}$$ and $$\hbox {IMG}_{\text {E+2W}},$$ which suffer slightly less from this problem and show less under-translation issues than other MNMT models. We note that the PBSMT model is expectedly resilient to the *missing words* error type. The main reason is perhaps the fact it implements a coverage mechanism within its decoding algorithm, i.e. in the decoding of a translation for a source sentence, by design each source word is translated once (Koehn 2010; Och and Ney 2004), which does not happen in a standard implementation of a greedy or beam search for NMT.

#### Incorrect words

In the incorrect words category, we note that multi-modal models in most cases outperform both baselines. The PBSMT baseline is the one that produces more incorrect translations units, including *mistranslation* and *incorrect form*, *extra words*, and *style*. It is also the PBSMT baseline that produces more *extra-words* (6), followed closely by model $$\hbox {IMG}_{\text {E}}$$ (5), being these two models the ones which produce translations with more repetitive, over-translated content. $$\hbox {IMG}_{\text {2W}},$$$$\hbox {IMG}_{\text {D}}$$ and the other multi-modal model combinations suffer less from that problem, but still present it considerably. Introducing some form of attention memory—making the model aware of its previous attention weights for the words generated in the previous time steps—is likely to improve these type of errors, as discussed by Tu et al. (2016). Finally, neither of the models, baselines and multi-modal, suffer much from *incorrect form* errors. Nevertheless, the PBSMT baseline is clearly the one with the worst results in the *incorrect words* error category, showing pronounced *mistranslation* errors, arguably some of the errors that impact the most in the perception of translation quality. We note that *mistranslation* is arguably the most damaging error type within this category, and that there is a clear trend of MNMT models performing considerably better than both text-only baselines, e.g. sometimes presenting less than half of the number of errors of the baselines.

#### Other error types

The PBSMT baseline clearly has pronounced out-of-vocabulary issues, derived from its lack of ability to extrapolate from a fixed set vocabulary by default. Its huge number of *unknown word* errors (a total of 35) has arguably a very strong negative impact in the perception of translation quality, and we stress that none of the other NMT systems, baseline and multi-modal, present any *unknown word* errors. This is a very important characteristic of these (neural) models, and it partially derives from the fact that all the data fed to them is always preprocessed into subwords (Sennrich et al. 2016). In preliminary experiments, Calixto (2017) trained PBSMT models with subwords instead of words to try to alleviate the amount of *unknown words* errors but found that translations consistently deteriorated (as measured by standard MT metrics, e.g. BLEU Papineni et al. 2002). For that reason, we decided to use sentences tokenised into words to train all PBSMT models discussed in this article.

#### Visual terms

Regarding the translation of visual terms, the PBSMT baseline is clearly the worst performing one, with 45 mistranslations and only 120 correct ones. The NMT baseline performs considerably better but is still the second worst with 37 mistranslations and 127 correct ones, a considerable improvement over the PBSMT baseline. Model $$\hbox {IMG}_{\text {E+D}}$$ is the one with the least number of *mistranslations* and *incorrect but interesting* translations of visual terms, with 27 translated units in these categories, and also with the highest number of *correct* visual terms translations, a total of 138 correct translations. It is closely followed by models $$\hbox {IMG}_{\text {2W+D}},$$$$\hbox {IMG}_{\text {E}},$$$$\hbox {IMG}_{\text {D}},$$ and $$\hbox {NMT}_{\text {SRC+IMG}},$$ with 137, 136, 136, 135 correctly translated units, respectively.

We note a clear trend here of MNMT models outperforming the text-only baselines when translating visual terms (model $\hbox {IMG}_{\text {2W}}$ perhaps less prominently so).

#### General trends

**Fig. 3:**
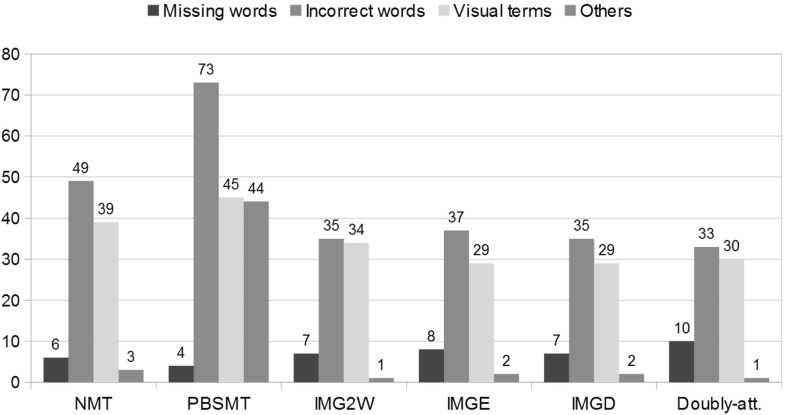
Number of errors different MT models (text-only baselines and multi-modal NMT models) make when translating 50 random sentences from the $\hbox {M30k}_{\text {T}}$ test set, split by error category. All models are trained on the original $\hbox {M30k}_{\text {T}}$ training set only

In Fig. 3, we show the number of errors that different baselines and MNMT models make, aggregated by error category. We note that MNMT models tend to produce less errors across all error categories, perhaps the only exception being the *missing words* errors, as already discussed.

#### Novel visual terms and other interesting examples

We now introduce and discuss some translations of novel visual terms generated by some multi-modal models, as well as other interesting examples. In general, all multi-modal models analysed generate a few novel visual terms, although these are not too frequent (a maximum of 6 examples in the 50 sentences analysed). MNMT models present a considerably larger amount of interesting/novel visual terms in comparison to the text-only baselines. Even though it is clear that many of the cues to translate these terms are already present in the textual portion of the training data, the fact that these show up considerably more frequently in the MNMT models show that the image is an important catalyst to generating so-called novel visual terms.

In Table 2, we show an example where neither the source German sentence nor the English reference translation contained the translated unit “paddling down”, although paddles are clearly visible in the image. Looking into the M30k training sentences, there are few examples where a sentence describing people in kayaks or kayaking also include the words “paddle” or “paddling”, so the models have not necessarily taken that information strictly from the image, although the image seems to have helped since neither the PBSMT nor the NMT baselines included these terms.

In Table 3, we draw attention to the example containing two novel visual terms of interest. In the two cases, neither the source German sentence nor the English reference translation contained the translated units “having fun” or “Mexican restaurant”, although both could have been inferred at least partially from the image. In this example, one of the novel visual terms, “having fun”, is also generated by the baseline NMT model, making it clear that at times what seems like a translation extracted exclusively from the image may have been learnt from the textual part of the training data. However, none of the two text-only NMT or PBSMT baselines translated “Mexikanischen Setting” into “Mexican restaurant”, but four out of the five multi-modal models did. The multi-modal models also had problems translating the German “trinkt Shots” (drinking shots). We observe translations such as “having drinks” ($\hbox {IMG}_{\text {2W}}$), which although not a novel translation is still a correct one, but also “drinking apples” ($\hbox {IMG}_{\text {E}}$), “drinking food” ($\hbox {IMG}_{\text {D}}$), and “drinking dishes” ($\hbox {IMG}_{\text {E+D}}$), which are clearly incorrect.

**Table 2:** Examples of translations for the example 219 in the M30k test set, where some translations involve novel visual terms (highlighted in bold-face)

Model	Translation
Source	zwei Männer fahren in Kajaks, eines orange und das andere blau, einen Fluss hinab
Reference	Two guys are kayaking, one orange one blue, down a creek
NMT	Two men are kayaking in orange, one in orange and the other in blue, going down a river
PBSMT	Two men are kayaks in an orange, blue, and the other in a river
$\hbox {IMG}_{\text {2W}}$	Two men are kayaking down a river in yellow kayak, orange and the other blue
$\hbox {IMG}_{\text {E}}$	Two men are kayaking through kayaks, one in orange and the other blue, are **paddling down** a river
$\hbox {IMG}_{\text {D}}$	Two men are kayaking in kayaks, one in orange and the other blue is going down a river
$\hbox {IMG}_{\text {2W+D}}$	Two men are kayaking, orange and the other blue, **paddling down** a river
$\hbox {IMG}_{\text {E+D}}$	Two men are kayaking in kayaks, one in orange and the other blue, is **paddling down** a river

**Table 3:** Examples of translations for the example 300 in the M30k test set, where some translations involve novel visual terms (highlighted in bold-face)

Model	Translation
Source	eine Gruppe junger Menschen trinkt Shots in einem Mexikanischen Setting
Reference	A group of young people take shots in a Mexican setting
NMT	A group of young people are **having fun** in an auditorium
PBSMT	A group of young people drinking at a Shots Mexikanischen Setting
$\hbox {IMG}_{\text {2W}}$	A group of young people having drinks in a **Mexican restaurant**
$\hbox {IMG}_{\text {E}}$	A group of young people drinking apples in a **Mexican restaurant**
$\hbox {IMG}_{\text {D}}$	A group of young people drinking food in a **Mexican restaurant**
$\hbox {IMG}_{\text {2W+D}}$	A group of young people **having fun** in a Mexican room
$\hbox {IMG}_{\text {E+D}}$	A group of young people drinking dishes in a **Mexican restaurant**

In Table 4, we show a simpler example that still demonstrates the strengths multi-modal models bring when translating visual terms. In this example, four out of five multi-modal models translate “Nonnen” (nuns) as “women”, whereas the other one translates it as “girl”, which is incorrect but still arguably better than the two baselines; the NMT model translates it as “men”, and the PBSMT baseline copied the source word “Nonnen” as is, i.e. it is an out-of-vocabulary word. This example showcases that the PBSMT baseline can still leave words untranslated, i.e. out-of-vocabulary, and a strong text-only NMT baseline can still make basic mistakes, even when translating simple sentences like this.

**Table 4:** Examples of translations for the example 720 in the M30k test set, where some translations involve novel visual terms (highlighted in bold-face)

Model	Translation
Source	zwei Nonnen posieren für ein Foto
Reference	Two nuns are posing for a picture
NMT	Two men pose for a picture
PBSMT	Two Nonnen posing for a picture
$\hbox {IMG}_{\text {2W}}$	two girls posing for a picture
$\hbox {IMG}_{\text {E}}$	Two **women** pose for a picture
$\hbox {IMG}_{\text {D}}$	Two **women** pose for a picture
$\hbox {IMG}_{\text {2W+D}}$	Two **women** pose for a picture
$\hbox {IMG}_{\text {E+D}}$	Two **women** pose for a picture

In Table 5, we show an interesting example. Here, the German source sentence is incorrect; it looks like it was incorrectly tokenized. One of the ways to fix sentence “ein Mann verwe ndet elektro nische Geräte” is to rewrite it as “ein Mann *verwendetelektronische* Geräte.” This is the German sentence which corresponds to the reference translation “a man is using electronic equipment.”

We note that the PBSMT model is unable to cope with these errors in the source sentence. Its translation simply left “verwe ndet elektro nische” untranslated, which has a clear negative impact in the subjective quality of the output. Nonetheless, all NMT models (including the baseline) have managed to translate “verwe ndet elektro nische” more or less accurately. The translation generated by the baseline NMT model mentions “a pair of equipment”, which is again wrong but conveys some of the meaning in the source. Most of the translations generated by the multi-modal models are better, with one translation in special. Model $\hbox {IMG}_{\text {E}}$ translates “verwe ndet elektro nische Geräte” (*is using electronic equipment*) as “is playing a DJ equipment”, which is surprisingly accurate, even though this information is clearly not in the source nor in the reference translation.

**Table 5:** Examples of translations for the example 339 in the M30k test set, where one translation includes novel visual terms (highlighted in bold-face)

Model	Translation
Source	ein Mann verwe ndet elektro nische Geräte
Reference	A man is using electronic equipment
NMT	A man is working with a pair of equipment
PBSMT	A man verwe ndet elektro nische equipment
$\hbox {IMG}_{\text {2W}}$	A man is working on some equipment
$\hbox {IMG}_{\text {E}}$	A man is playing a **DJ equipment**
$\hbox {IMG}_{\text {D}}$	A man is working on welding equipment
$\hbox {IMG}_{\text {2W+D}}$	A man is working on some equipment
$\hbox {IMG}_{\text {E+D}}$	A man is playing a piece of equipment

We again looked into the M30k training sentences, and there are a few examples where sentences describe “DJ” or “DJ equipment”. That means that the models have not necessarily taken that information strictly from the image since they have seen these in a few training sentences, although the image seems to have helped since neither the PBSMT nor the NMT baselines included these terms.

#### Final remarks

In general, MNMT models present considerably less errors than both text-only baselines, NMT and PBSMT. MNMT models that use global visual features fare particularly well when translating image descriptions. Even tough model $\hbox {NMT}_{\text {SRC+IMG}}$ still presents a number of errors comparable to other MNMT models that use global image features, it uses more parameters than its counterparts.

We note that the most damaging type of error we evaluate to the perceived quality of a translation is the *mistranslation* in the *incorrect words* category, and the *mistranslations* in the *visual terms* category. These errors were, on average, drastically diminished in all MNMT models compared to both text-only NMT and PBSMT baselines, i.e. *mistranslation* errors decreased from 41/52 (NMT/PBSMT) to 23–29 depending on the MNMT model, and *mistranslations* of visual terms decreased from 37/45 (NMT/PBSMT) to 27–32 errors, again depending on the MNMT model.

These are all strong findings that support our initial intuition that multi-modal models are not only better than text-only models at translating image descriptions according to automatic MT metrics, but also according to a targeted error analysis where different error types are considered. Crucially, we demonstrate that multi-modal models reduce not only errors related to the translation of visual terms, but also considerably reduce more general errors, i.e. *incorrect words* category. This is in itself an interesting finding, since it implies that adding multi-modal, visual signals is helpful not only in the obvious situations where we wish to translate visual terms. On the contrary, our error analysis indicates that improvements are distributed across visual and non-visual portions of the text, which is a surprising collateral impact.

## Conclusions and future work

In this work, we conducted an extensive error analysis of the translations generated by different baselines, a PBSMT model and a standard attention-based NMT baseline, and by MNMT models that incorporate images into state-of-the-art attention-based NMT by using images as words in the source sentence, to initialise the encoder’s hidden state, as additional data in the initialisation of the decoder’s hidden state, and by means of an additional independent visual attention mechanism. The intuition behind our effort is to assess how humans perceive translations generated by models that make use of images to visually *ground* translations, and in principle increase translation quality by doing so.

We corroborate findings obtained using standard automatic MT evaluation metrics in the original publications of the models by Calixto and Liu (2017) and Calixto et al. (2017), and demonstrate with an extensive error analysis that adding global and local image features into NMT significantly improves the translations of image descriptions compared to both text-only NMT and PBSMT models. When adding local (i.e. spatial) visual features as in the doubly-attentive MNMT model of Calixto et al. (2017), the positive impact is comparable to those obtained with the simpler MNMT models that use only global image features, but model $\hbox {NMT}_{\text {SRC+IMG}}$ has more parameters. We conjecture that the reason for this might be that model $\hbox {NMT}_{\text {SRC+IMG}}$ has two *independent* attention mechanisms, one visual and one textual, whereas the connection between the image description in the source language and parts of the image is as strong as that between (parts of) the image and its description in the target language. An architecture where the two attention mechanisms are independent would in theory require more data in order to estimate strong mappings from target words to parts of an image, but perhaps if one could use instead some form of *hierarchical attention*, e.g. as proposed by Libovický and Helcl (2017), that might be a way to address this issue and learn a better model with less data.

Error types most damaging to the perception of translation quality, arguably the *wrong sense* error in the *incorrect words* category, and the *spurious* translations in the *visual words* category, are among the ones where there are greater gains comparing MNMT models to the text-only baselines.

In future work, we will use the lessons learnt in this analysis and will investigate how to model reasoning with natural language generation models grounded on multi-modal representations.

## Footnotes

1 This work is partially derived from the first author’s Ph.D. Thesis (Calixto 2017).

2 Instead, if we used the ResNet-50 network (He et al. 2015) to extract local and global features for one same image, the local features (i.e., layer res4f) would consist of a $196 \times 512$D matrix, whereas global features (i.e., layer FC1000) would consist of a 1024D vector, an increase of $98 \times$.

3 For more details, see https://github.com/nyu-dl/dl4mt-tutorial/blob/master/docs/cgru.pdf.
